# Eagle Syndrome, A Third Variant: Giant Styloid Processes, Pseudoarthrosis, and Unilateral Facial Spasms Progressing to Facial Paralysis

**DOI:** 10.1155/crot/2810812

**Published:** 2025-08-04

**Authors:** Claire J. Hoffman, Paul M. Bunch, Nelson H. May, Eric M. Kraus, Christopher A. Sullivan

**Affiliations:** ^1^Department of Otolaryngology–Head and Neck Surgery, Wake Forest University School of Medicine, Winston-Salem, North Carolina, USA; ^2^Department of Radiology, Division of Neuroradiology, Wake Forest University School of Medicine, Winston-Salem, North Carolina, USA; ^3^Department of Otolaryngology–Head and Neck Surgery, Wellstar Kennestone Hospital, Marietta, Georgia, USA

## Abstract

We describe a third variant of Eagle syndrome characterized by (1) neck pain (2) giant, hypertrophied styloid processes, (3) mobile styloid process due to pseudoarthrosis, and (4) combined facial nerve compression/entrapment leading to facial spasms and paralysis. Our patient presented with symptoms of Eagle syndrome, bilateral giant styloid processes, and left facial spasms progressing to left facial paresis/paralysis. CT findings included a pseudoarthrosis of the base of a giant left styloid process and overgrowth of bone superior to the styloid base. The mobile styloid process resulted in compression of the facial nerve exiting the stylomastoid foramen and facial spasms with head rotation to the left. Bony overgrowth superior to the pseudoarthrosis led to stenosis of the facial canal distal mastoid segment. Along with compression from the styloid, nerve entrapment contributed to facial paresis that progressed to facial paralysis. Left styloidectomy was performed to alleviate impingement on the facial nerve, and left canal wall-up tympanomastoidectomy with facial nerve decompression was performed to alleviate entrapment caused by bony overgrowth. Two months postoperatively, the patient's left facial paralysis improved to mild left eyelid lag without signs or symptoms of ocular exposure. The patient developed delayed first-bite syndrome successfully treated with amitriptyline. She did not report postoperative dysgeusia. Our patient's left facial paresis was initially attributed to viral neuritis, and the possibility of Eagle syndrome was not initially considered. The relative rarity and varied presentations of Eagle syndrome often create a diagnostic challenge, especially for patients with progressive symptoms. Our patient's presentation emphasizes the importance of a thorough head and neck evaluation of any patient presenting with head and neck signs or symptoms persisting for longer than 3 weeks as well as awareness of what we believe is the first description of a third variant of Eagle syndrome.

## 1. Introduction

Eagle syndrome is a complex clinical condition that can present with a range of symptoms and findings. Most patients report neck pain that is caused by a congenitally elongated or damaged styloid process that can exert a mass effect on nearby structures, including the facial nerve.

Eagle syndrome was initially described in 1937 as “*stylalgia*” [1]. Since the initial description, there has been progress in understanding, categorizing, and treating the rare disorder. Diagnosing Eagle syndrome requires a high index of suspicion.

Historically, two different variants of Eagle syndrome have been described—classic and carotid. The classic variant is characterized by pain and dysphagia. The carotid variant is characterized by pain and cerebral ischemia, internal carotid artery dissection, or syncope [2, 3]. However, both variants can result in compression of the facial, glossopharyngeal, and vagus nerves [4]. For those with severe symptomatic disease, surgical management is the standard of care. We describe a third variant that includes direct facial nerve compression by a mobile styloid process and facial nerve entrapment within the distal vertical descending facial canal secondary to bony overgrowth from the underlying disease process.

## 2. Case Report

### 2.1. History

A 42-year-old female was referred to our outpatient head and neck clinic with a history of hypertension, temporomandibular joint disorders managed with diet and mouth guard through dentistry, and possible Eagle syndrome. Her chief complaint was pain located inferior to her left external ear, in her cheek, and in her mouth. She reported left facial spasms with head turns to the left and progressive left facial paresis/paralysis. She did not report dysgeusia. She denied any facial/neck trauma. She was administering artificial tears to her left eye and denied any symptoms of exposure keratitis or change in vision.

Prior to our evaluation, she had been diagnosed elsewhere as having left Bell's Palsy and had been treated with oral steroids and antiviral medications for 2 weeks without resolution of symptoms. Her left facial paresis began in the lower division and progressed from inferior to superior. Importantly, she developed left facial spasms involving her left upper lip, cheek, and eyelid with head rotation to the left. The left facial paresis progressed to complete left facial paralysis.

### 2.2. Physical Examination

On physical exam, she had complete left facial paralysis, House Brackmann 6, with 1 mm of left lagophthalmos and no evidence of exposure keratitis. Right facial function was intact and normal. Both tympanic membranes were clear and mobile. Both middle ear spaces were well aerated without evidence of infection or cholesteatoma. The neck was supple without cervical lymphadenopathy, thyromegaly, or palpable masses. Parotid glands were soft and without any palpable masses. There were no carotid bruits. With the exception of the left facial nerve, cranial Nerves II through XII were intact.

### 2.3. Audiometric Testing

On comprehensive audiometric testing, hearing was normal in both ears.

### 2.4. Imaging

**Figure 1:**
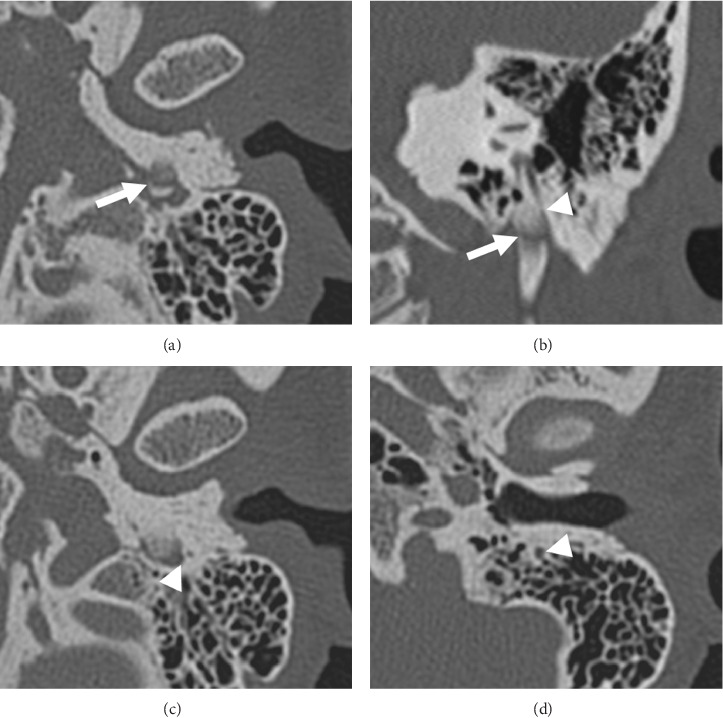
(a) Axial left temporal bone CT image at the level of the left stylomastoid foramen demonstrates an abnormal lucency suggestive of pseudoarthrosis through the base of the left styloid process (arrow). (b) Coronal left temporal bone CT image at the same level confirms the suspected pseudoarthrosis (arrow) and demonstrates apparent narrowing of the mastoid segment of the left facial nerve canal (arrowhead) due to osseous overgrowth of the base of the left styloid process at a level superior to the pseudoarthrosis. (c) Axial left temporal bone CT image at the level of the base of the left styloid process confirms narrowing of the distal mastoid segment of the left facial nerve canal (arrowhead) due to the overgrown left styloid process base. (d) For comparison, the axial left temporal bone CT image at the level of the left external auditory canal demonstrates the normal appearance of the mastoid segment of the left facial nerve canal (arrowhead).

**Figure 2:**
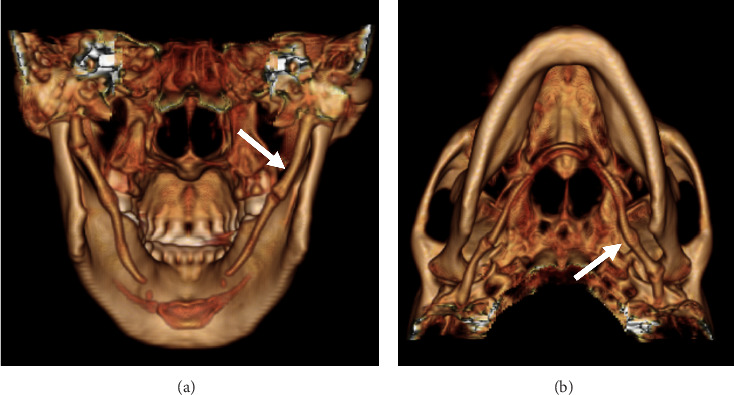
(a, b) 3D volume-rendered images from posterior (a) and inferior (b) perspectives demonstrate elongation of both right and left styloid processes with ossification of the stylohyoid ligaments, each process measuring over 7 cm in length (arrows pointing to the giant left styloid process).

**Figure 3:**
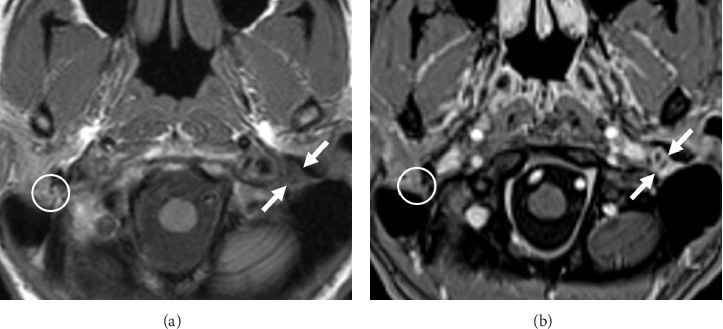
(a, b) Axial MRI T1-weighted images acquired before (a) and after (b) administration of gadolinium contrast demonstrate abnormal T1 hypointense, enhancing soft tissue (Arrows A and B) effacing the fat of the left stylomastoid foramen. The normal appearance of the right stylomastoid is marked (Circles A and B) for comparison. In conjunction with the CT scan findings, the enhancing soft tissue was considered most likely to represent inflammation and granulation tissue forming in reaction to pathologic motion at the left styloid process pseudoarthrosis.

CT imaging (Figures 1(a), 1(b), 1(c), and 1(d)) with 3D-volume rendering (Figures 2(a) and 2(b)) and MR imaging (Figures 3(a) and 3(b)) were performed. Imaging revealed giant styloid processes, a pseudoarthrosis of the base of the left styloid process, and overgrowth of the base of the left styloid at a level superior to the pseudoarthrosis resulting in partial stenosis of the distal mastoid segment of the facial canal.

### 2.5. Diagnosis

Based on the imaging findings, the patient was diagnosed as having Eagle syndrome with pseudoarthrosis of a giant left styloid process and pathologic motion at its base. It was surmised that the mobile styloid was causing direct compression of the left facial nerve at the level of the stylomastoid foramen manifested as facial spasms with leftward head rotation. In addition, bony overgrowth superior to the base of the styloid was resulting in entrapment of the facial nerve in the distal vertical descending mastoid segment. The combination of both pathologies contributed to the progression of left facial paresis to paralysis. Operative intervention was offered.

### 2.6. Operative Procedure

The patient underwent synchronous procedures consisting of an uncomplicated left canal wall-up tympanomastoidectomy with facial nerve decompression and a left styloidectomy. This required collaborative effort between the otologist and head and neck surgeon to provide the best outcome for the patient.

### 2.7. Facial Nerve Decompression

**Figure 4:**
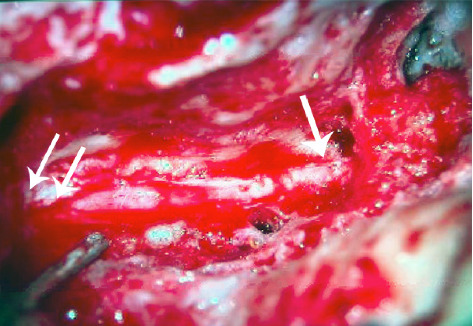
Left transmastoid facial nerve decompression. The facial nerve was exposed from the second genu proximally (single arrow) to the stylomastoid foramen distally (double arrow). The lateral 180° of the vertical descending segment of the facial nerve canal was removed, and the epineurium was slit with a #375910 microknife to decompress the nerve.

The left facial nerve was identified through a postauricular, transmastoid approach along its usual course from the second genu proximally to the stylomastoid foramen distally. The vertical descending course of the nerve was consistent with the preoperative imaging. Bone was removed from the nerve laterally for 180° from the second genu to the stylomastoid foramen. Once exposed, the epineurium was incised to allow decompression of the facial nerve (Figure 4). The nerve was noted to be particularly edematous just superior to the stylomastoid foramen and initially did not stimulate with a facial nerve stimulator at 2 mA prior to slitting of the epineurium. Following decompression, the nerve stimulated proximally, just inferior to the second genu at 0.1 mA, and stimulated distally at the stylomastoid foramen at 0.15 mA.

### 2.8. Styloidectomy

**Figure 5:**
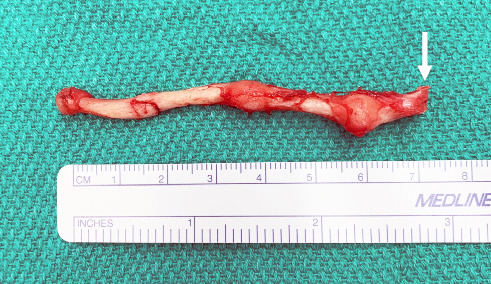
Giant left styloid process specimen measuring greater than 7 cm (arrow pointing to the proximal styloid process).

A left extended, external neck incision was joined to the postauricular incision in preparation for a synchronous left styloidectomy. The main trunk of the left facial nerve was exposed distal to the stylomastoid foramen. With the main trunk of the left facial nerve well identified and well protected at its exit from the stylomastoid foreman, the giant left styloid process, measuring greater than 7 cm in length, was excised (Figure 5). A suction drain was inserted. There were no complications.

### 2.9. Postoperative Course

One week postoperatively, the patient regained resting left facial tone, but her face remained paretic upon voluntary motion. She did exhibit slightly more power in her left muscles of facial expression than she did preoperatively.

One month postoperatively, the patient reported moderate-to-severe pain in the region of the left parotid gland, left temporomandibular region, and left external auditory canal associated with initial bites of food consistent with “*first bite syndrome*.” First bite syndrome refers to severe pain, typically in the preauricular region, with the first bite of food. First bite syndrome may occur after upper neck operations and is believed to result from loss of sympathetic innervation to the parotid gland [5]. There was no evidence of hypertrophic scarring or keloid formation. The left facial paralysis eventually improved to only slight eyeblink weakness of the left upper eyelid.

Seven weeks following operation, left facial nerve function was essentially normal in all branches except for mild left eyelid lag. The patient reported that when she closed her eyelids tightly, she experienced synkinesis resulting in motion of her upper lip. Amitriptyline was initiated for the first bite syndrome.

Over time, the patient's left facial tone and power returned nearly to her full, premorbid function. Synkinesis resolved, and first-bite syndrome was managed with diet only. The patient's initial facial, ear, and neck pain resolved. The patient remained asymptomatic from her giant right styloid process without evidence of pseudoarthrosis.

## 3. Discussion

Eagle syndrome is characterized by neck pain and other symptoms caused by an elongated styloid process and/or an ossified stylohyoid ligament. Symptomatic presentation is critical in entertaining the diagnosis, which requires a high index of suspicion. CT imaging best characterizes the size and morphology of the styloid processes and stylohyoid ligaments.

A normal styloid process measures between 2.5 and 3 cm in length. In symptomatic patients, the styloid process is often longer than 3 cm in length and is classified as elongated [6]. In our patient, the styloid process measured over 7 cm in length.

An elongated styloid process may exert positional pressure on surrounding structures, including the facial nerve, glossopharyngeal nerve, vagus nerve, jugular vein, and carotid artery. Direct pressure may lead to pulsatile tinnitus due to jugular vein compression and may cause neck pain that may be accompanied by a variety of additional symptoms. In our case, both styloid processes were elongated and hypertrophied, although only the left side became symptomatic due to the unusual pseudoarthrosis, bony overgrowth, and facial nerve compression/entrapment.

We note that it would be unusual for Eagle syndrome alone to lead to facial spasms and progressive facial paralysis. However, as this atypical case illustrates, the patient developed what we believe is a third variant of Eagle syndrome characterized by (1) neck pain, (2) giant, hypertrophied styloid processes, (3) a mobile styloid process due to pseudoarthrosis causing direct facial nerve compression and facial spasms with head rotation, and (4) progressive ipsilateral facial paresis/paralysis due to bony overgrowth superior to the pseudoarthrosis leading to partial stenosis of the distal vertical descending portion of the facial canal with nerve entrapment.

A thorough history and physical examination along with a strong index of clinical suspicion are necessary to diagnose Eagle syndrome in a timely manner. Our patient was initially diagnosed as having Bell's Palsy and viral neuritis which did contribute to delay in making the correct diagnosis of an atypical variant of Eagle syndrome. While prompt decompression provides the best chance of a favorable outcome, this patient was still able to find symptom relief.

Even with the delay in presentation, we would have offered the patient a styloidectomy and facial nerve decompression even if she would have presented at a much later date. One author (EK) has decompressed the facial nerve of patients with idiopathic facial paralysis up to 1 year following onset with meaningful improvement (e.g., House Brackmann 6 to House Brackmann 3 status). We believe that some resting facial tone and power are better than none.

Appropriate imaging, such as computerized tomography scanning and MRI, assists the clinical diagnosis of Eagle syndrome and helps to guide operative intervention. In addition to characterizing the morphology of the styloid process and the stylohyoid ligament, imaging informs whether there is evidence of previous trauma, styloid process pseudoarthrosis at the base of the skull, fracture, or compression of the adjacent neural and vascular structures.

In addition, during evaluation of presumed Eagle syndrome, it is important to rule out chronic ear and parotid disease as well as to obtain an accurate evaluation of the styloid process. In our case, preoperative imaging also demonstrated a pseudoarthrosis at the base of the enlarged left styloid process and bony overgrowth with associated narrowing of the distal descending segment of the left facial nerve canal.

Given the imaging findings of pseudoarthrosis of the base of the styloid, mechanical compression of the left facial nerve was implicated as the cause of the patient's left facial spasms, as the spams occurred with left head turns. The facial paresis progressed eventually to complete paralysis. We do believe that mechanical compression of the nerve was due primarily to the partially mobile giant styloid process. Based on the time course, CT images, and intraoperative findings, the partial stenosis of the distal vertical descending segment of the facial canal was felt to be playing a secondary role. If partial stenosis of the facial canal alone was the etiology of the facial paresis, we do not believe that head turning would have caused facial spasms, and the time course would have been much slower.

Complete surgical treatment of her condition required synchronous facial nerve decompression and styloidectomy. Neither procedure alone would have been sufficient to relieve the mechanical nerve compression and entrapment. Based on CT and MRI imaging clearly demonstrating significant hypertrophy of the proximal styloid at the skull base as well as narrowing of the distal facial canal, it was prudent to both decompress the nerve and to clearly expose the nerve distal to the stylomastoid foramen in order for the styloidectomy to be performed as safely as possible with the nerve clearly identified. We believe that a team approach is both optimum and effective.

In these patients, postoperative surveillance of facial nerve function is recommended. Distinguishing between the return of resting facial tone and the return of facial power under voluntary control is important, as facial tone usually returns before facial power. Both functions are extremely important to the patient.

In the setting of bilateral styloid process hypertrophy and elongation, the asymptomatic, contralateral styloid process should be assessed and monitored. The patient should be made aware of return precautions, as future symptoms may occur. Furthermore, in any patient with facial paresis or paralysis, care should be taken to protect the globe and cornea with lubricants, artificial tears, and oblique eyelid taping until the lagophthalmos resolves and complete eyelid closure function returns.

Finally, the patient's presentation and associated time to diagnosis raise the importance of using our “*3-week rule*” as a guide for evaluating head and neck symptoms that are unilateral and do not resolve spontaneously. Specifically, head and neck symptoms that do not resolve spontaneously or respond to conservative medical intervention within 3 weeks, especially unilateral signs and/or symptoms, merit further investigation and work-up. The work-up should include imaging studies (e.g., neck CT and MRI), to evaluate for any underlying pathology that may be accounting for the patient's persistent symptoms. We feel strongly that it is imperative to examine the patient on the “*outside*” as well as on the “*inside.*”

## Data Availability

Data sharing is not applicable to this article as no datasets were generated or analyzed during the current study.
